# CHANGING NURSES’ BEHAVIOR TOWARD NUTRITIONAL CARE WITH A SNACK-SIZED LEARNING INTERVENTION

**DOI:** 10.1093/geroni/igz038.182

**Published:** 2019-11-08

**Authors:** Debbie Ten Cate, Jeroen Dikken, Roelof Ettema, Lisette Schoonhoven, Marieke J Schuurmans

**Affiliations:** 1 University of Applied Sciences Utrecht, Utrecht, Netherlands; 2 The Hague University of Applied Sciences, Den Haag, Zuid-Holland, Netherlands; 3 University of Applied Sciences Utrecht, Utrecht, Utrecht, Netherlands; 4 Julius Center for Health Sciences and Primary Care, University Medical Center Utrecht, Utrecht, Utrecht, Netherlands; 5 Education Center, UMC Utrecht Academy, University Medical Center Utrecht, Utrecht, Utrecht, Netherlands

## Abstract

Nurses play an important role in the prevention and treatment of malnutrition in older adults. However, research shows that nurses lack the motivation to give adequate nutritional care. In order to change this motivation a learning intervention about nutrition in older adults targeted at nurses would be desirable. The aim of this study was to assess the development, validation, and reliability of a learning intervention about nutrition in older adults. The results show that this learning intervention has a good construct and content validity, and is psychometrically sound. Questions of the learning intervention can be presented at once or in a snack-sized way, where the questions are presented over a period of time. This learning intervention can be used for developers of similar interventions, and as part of educating programs for nursing professionals and nursing students about nutritional care for older adults.

